# improvement of peripheral visual discrimination through mental imagery

**DOI:** 10.1016/j.isci.2026.116437

**Published:** 2026-06-17

**Authors:** Fazilet Zeynep Yildirim-Keles, Rahel Aschwanden, Bilge Sayim

**Affiliations:** 1 Department of Psychology, Boğaziçi University, Istanbul 34342, Türkiye; 2 Institute of Psychology, University of Bern, Fabrikstrasse 8, 3012 Bern, Switzerland; 3 Institute of Marketing and Management, University of Bern, Engehaldenstrasse 4, 3012 Bern, Switzerland; 4 Laboratoire de Sciences Cognitives et Psycholinguistique, Département D’Études Cognitives, École Normale Supérieure, PSL University, CNRS, Paris, France

**Keywords:** biological sciences

## Abstract

Visual perception is fundamentally limited by crowding, the impairment of object recognition by nearby elements. Previous work has shown that crowding can be alleviated through long-range grouping mechanisms: Visual discrimination improved when a crowded peripheral target matched the centrally presented item. This study investigated whether mental imagery can alleviate crowding and improve peripheral discrimination. Consistent with our hypothesis, we found that target discrimination improved when observers imagined an item matching the crowded peripheral target. Importantly, this facilitation did not occur when imagining a different item or an item matching in semantic content only. These results suggest that imagery engages detailed visual representations that can interact with degraded peripheral visual input to enhance perceptual discrimination. Our findings have important implications for the use of mental imagery to improve peripheral vision in everyday contexts, sports, and clinical settings.

## Highlights

• Mental imagery reduced visual crowding, improving peripheral discrimination
• Discrimination improved for shape-matched, not semantically matched targets
• Imagery enhanced degraded signals via shape-based grouping
• Mental imagery can enhance visual perception

## Graphical abstract

**Figure:**
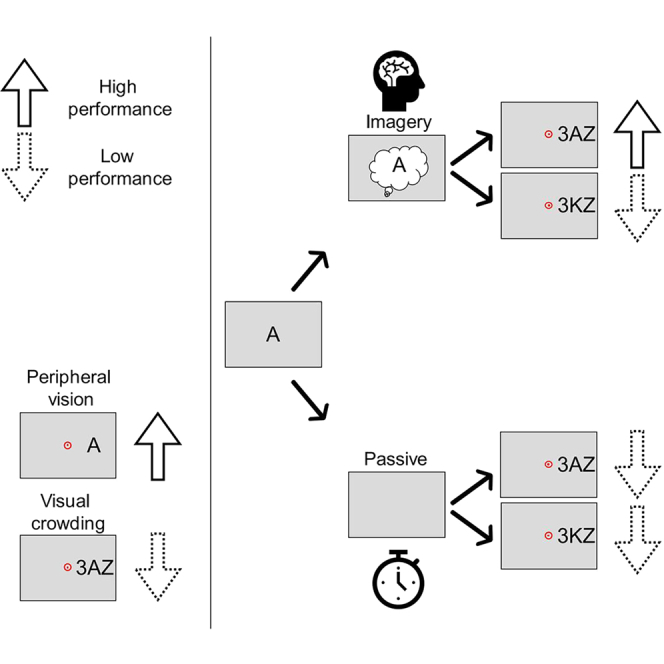

## Introduction

Visual mental imagery (VMI) refers to the process of creating mental “images” or “pictures” in the mind’s eye without the presence of actual physical stimuli. It has been shown to enhance performance in visually demanding tasks. For example, it was shown that visual imagery can yield perceptual learning.^1^ In a collinear facilitation paradigm, imagining the flankers has been shown to improve target detection, comparable to the improvement observed when the flankers were physically present.^2^ Farah^3^ showed that imagery of a letter that matched a peripheral target improved target identification. These findings illustrate how VMI can influence early visual processing. Indeed, research has demonstrated that VMI operates through mechanisms similar to those used in actual perception, engaging neural pathways that overlap with those activated by real visual stimuli.^4^^,^^5^^,^^6^^,^^7^ For example, studies have shown that VMI may affect sensory perception similar to real sensory stimuli, leading to effects such as improved visual awareness during binocular rivalry,^8^ faster detection of stimuli that match the imagined content during visual search tasks,^9^ and the integration with perceptual input to induce multisensory illusions.^10^ Given this capacity, VMI might also improve performance in basic visual tasks, such as mitigating the effects of visual crowding. Importantly, such influences do not override sensory input, but may instead bias or sharpen the interpretation of ambiguous visual signals through interactions between early visual representations and higher-level processes.

**Figure 1:**
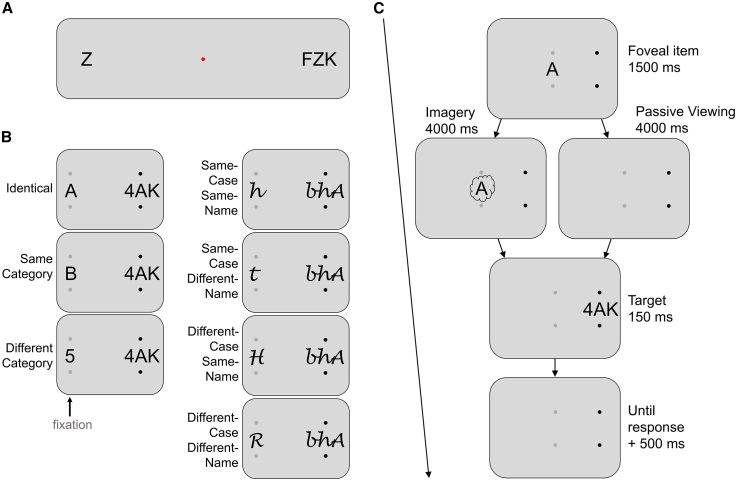
Demonstration of the visual crowding effect, the stimuli, and the procedure (A), the stimuli (B), and the procedure (C). (A) In visual crowding, identification of a peripheral target deteriorates when the target is surrounded by flankers: When fixating on the central red dot, identification of the isolated letter (left) is usually more accurate than the identification of the central, flanked letter. (B) Foveal-item categories in Experiment 1 (left) and 2 (right). (C) Illustration of a sequence of a trial in Experiment 1. The same procedure was used in Experiment 2. Following the offset of the foveal item, observers either actively visualized it (Imagery block) or refrained from doing so (Passive Viewing block). In both conditions, they were instructed to maintain fixation in the center between the gray dots. The black dots indicated the target location throughout the trial. The target appeared 4000 ms after the offset of the foveal item. Note that stimuli are not drawn to scale.

In visual crowding, the discrimination of objects deteriorates when they are surrounded by other items.^11^^,^^12^^,^^13^^,^^14^^,^^15^^,^^16^^,^^17^^,^^18^ For instance, when presented with a target letter in the visual periphery, recognition deteriorates when adding adjacent letters (flankers) (Figure 1A). Crowding is a fundamental limit to visual perception and has significant implications for various aspects of life, including reading,^19^ driving,^20^ and face recognition.^21^ Crowding not only impacts everyday activities, such as reading and object recognition, but also increases with age, contributing to slower reading in older adults,^22^ and tends to be stronger in a range of medical conditions that affect a substantial portion of the population, including amblyopia,^23^ macular degeneration,^24^ glaucoma,^25^^,^^26^ and dyslexia.^27^^,^^28^ Consequently, understanding the mechanisms underlying crowding^13^^,^^29^^,^^30^^,^^31^^,^^32^^,^^33^ and finding ways to alleviate its effects^34^^,^^35^^,^^36^^,^^37^ have been the focus of extensive research efforts. In the present study, we investigate the extent to which visual imagery can reduce visual crowding.

Building on earlier research that highlights the importance of interactions between peripheral and foveal inputs,^15^^,^^38^^,^^39^ we hypothesized that VMI could mitigate the effects of visual crowding by leveraging these interactions. Specifically, studies have shown that when a peripheral stimulus was identical to (“matched”) a foveal item, recognition and discrimination of the peripheral stimulus improved.^15^^,^^38^^,^^39^ This has been shown for both targets presented in isolation^39^ and in clutter (“crowded”;^15^). Different mechanisms have been proposed to account for this improvement. One proposed mechanism is feedback to the foveal cortex, in which fine-grained spatial resolution neurons in the foveal cortex are recruited to process extra-foveal shape information.^40^^,^^41^ Subsequent work provided psychophysical evidence for a temporally flexible feedback signal to the foveal cortex that can be disrupted or facilitated by foveal stimulation.^42^ More recent theoretical and empirical work further supports the idea that top-down feedback preferentially targets and enhances foveal representations, allowing peripheral stimuli to recruit foveal cortex to resolve ambiguous object information.^43^^,^^44^

Another proposed explanation is grouping, suggesting that shape-based grouping between identical foveal and peripheral items improves performance.^15^ Based on these findings, we investigated whether imagining a central item identical to the target would enhance peripheral target discrimination. To anticipate our results, we found significantly better target discriminability when participants imagined an identical central item compared to when they imagined a different central item or did not imagine the central item. These results suggest that visual imagery can mitigate the deleterious influence of neighboring elements in basic spatial vision.

## Results

### Experiment 1: Improving discrimination by visual imagery

In Experiment 1, we investigated whether imagining an item (a letter or a digit) at the fovea could improve the discrimination of a crowded target in the periphery. Stimuli consisted of letters and digits of Arial font, approximately 1° in size and located at 8° eccentricity. Specifically, the target and the flankers consisted of the letters “A, B, F, G, K, P, R, Y” and the digits “2, 3, 4, 5, 6, 7, 8, 9.” The foveal item was either a letter or a digit. It was either identical to the target (e.g., A/A or 3/3, referred to as “Identical”), different but of the same category as the target (e.g., A/B or 3/4, referred to as “Same Category”), or from a different category than the target (e.g., A/3, referred to as “Different Category”) (Figure 1B). Participants were instructed to imagine the foveal item in *imagery* blocks and to ignore it in *passive viewing* blocks (Figure 1C). Targets were either flanked by a letter and a digit on the left and right sides (i.e., Imagery and Passive Viewing blocks) or presented in isolation without a foveal item (i.e., Baseline block). Importantly, we ensured that targets were viewed peripherally using eye tracking: Trials in which gaze deviated more than 2° from central fixation were excluded. In all blocks, participants indicated whether the target was a letter or a digit. A second task in which participants rated the similarity of the foveal imagined item and the target on a scale from 1 (not similar at all) to 3 (very similar) was used to assure attention to both the foveal item and the target (results not reported here). The similarity-rating task ensured comparable task relevance of the foveal item in Imagery and Passive Viewing conditions.

**Figure 2:**
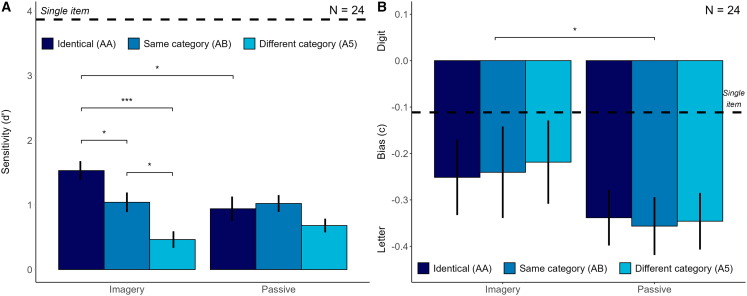
Results of Experiment 1 Sensitivity (A) and bias (B) results are shown across Imagery and Passive Viewing conditions for each foveal item category in Experiment 1. Error bars represent standard errors of the mean. The dashed horizontal line indicates the average d′ and c for the single-item (i.e., Baseline) condition, respectively. Asterisks denote significant pairwise comparisons between conditions: ∗*p* <0 .05, ∗∗*p* <0 .01, ∗∗∗*p* <0 .001, and ∗∗∗∗*p* <0 .0001. Note that, as the interaction effect was significant, comparisons were made within each imagery condition across categories and within each category across imagery conditions, focusing on theoretically relevant contrasts.

Figure 2 displays the results of Experiment 1. We calculated d′ (discriminability) scores separately for each foveal item category (Identical, Same Category, and Different Category) and imagery condition (Imagery, Passive Viewing, and Baseline). To assess the occurrence of crowding, we compared the Baseline to the Imagery and the Passive Viewing conditions (collapsed across category) using paired Wilcoxon tests with Holm correction. We found significantly higher d prime values in the Baseline condition (M = 3.87, SE = 0.10) compared to the Imagery (M = 1.01, SE = 0.10, *p* < 0.0001, *r* = 0.88) and Passive Viewing (M = 0.88, SE = 0.08, *p* < 0.0001, *r* = 0.88) conditions. These results confirm the presence of crowding, with lower discriminability in both the Imagery and Passive Viewing conditions compared to the Baseline condition. The Baseline condition did not include a foveal item, so the effect of flankers alone was not completely isolated; however, the large differences between conditions (i.e., Baseline d′ = 3.87 vs. Passive Viewing d′ = 0.88 or Imagery d′ = 1.01) suggest that any influence of a foveal item in the Baseline condition would be negligible.

A linear mixed-effects model (LMM) with fixed effects of foveal item category (Identical, Same Category, Different Category), imagery condition (Imagery, Passive Viewing), and their interaction, as well as a random intercept for participants (marginal *R*^2^ = 0.19; conditional *R*^2^ = 0.33), revealed a significant main effect of foveal item category (χ^2^(2) = 27.83, *p* < 0.0001). The main effect of the imagery condition was not significant (χ^2^(1) = 1.54, *p* = 0.21). There was a significant interaction between foveal item category and imagery condition (χ^2^(2) = 10.40, *p* = 0.006).

Specifically, in the Imagery condition, discriminability was significantly higher in the Identical foveal item compared to Same Category (mean difference (MD) = 0.49 ± 0.15, *t*(125) = 2.74, *p* = 0.04, *d* = 0.78) and Different Category items (MD = 1.07 ± 0.18, *t*(125) = 4.57, *p* = 0.0001, *d* = 1.69). Discriminability was also significantly higher in the Same Category compared to the Different Category items (MD = 0.58 ± 0.20, *t*(125) = 3.0, *p* = 0.02, *d* = 0.91). In contrast, in the Passive Viewing condition, there was no significant difference in discriminability scores between the Identical and the two other foveal item categories (Identical and Different Category: MD = 0.26 ± 0.24, *t*(125) = 1.40, *p* = 0.66, *d* = 0.41; Identical and Same Category: MD = −0.08 ± 0.24, *t*(125) = −0.44, *p* = 1.0, *d* = 0.13). Similarly, there was no significant difference between the Same Category and the Different Category items (MD = −0.34 ± 0.16, *t*(125) = −1.83, *p* = 0.35, *d* = 0.54).

Comparing discriminability in the Identical foveal item category between Imagery and Passive Viewing revealed higher scores in the Imagery condition (MD = 0.59 ± 0.18, *t*(125) = 3.17, *p* = 0.02, *d* = 0.94), while no such difference was observed for the Same Category (MD = 0.02 ± 0.18, *t*(125) = 0.10, *p* = 1.0, *d* = 0.029) and Different Category (MD = −0.22 ± 0.15, *t*(125) = −1.17, *p* = 0.74, *d* = 0.34) foveal items. A Wilcoxon test showed that discriminability was significantly higher in the Imagery-Identical condition compared to the Passive Viewing condition (collapsed across categories) (MD = 0.65 ± 0.12, *p* < 0.0001, *r* = 0.81). These findings suggest that imagery of an identical item at fixation improved the discrimination of crowded peripheral targets. Without visual imagery, foveal items did not modulate performance. We note that while some post-hoc *p* values (e.g., 0.04, 0.02) do not exceed the 0.01 level, the Imagery-Identical benefit is robust with large effect sizes (*d* = 0.78–1.69).

We also analyzed the proportion of responses consistent with the foveal item’s category separately for foveal-target congruent and incongruent trials to test whether the observed effects could be explained by a foveal-response strategy. The foveal-consistent responses were high when the foveal item and peripheral target belonged to the same category (congruent; between 0.76 and 0.66 ± SD 0.15–0.11), but were substantially reduced on incongruent trials (between 0.42 and 0.38 ± 0.10–0.09). This pattern indicates that participants did not simply default to reporting the foveal category when peripheral discrimination was difficult. Instead, response tendencies were systematically modulated by the relationship between the foveal item and the peripheral target.

We calculated c (bias) scores separately for each foveal item category and imagery condition. Bias in all conditions was negative, showing a tendency to report “letter”. Bias in the Baseline (M = −0.11, SE = 0.03) condition was smaller than in the Passive Viewing (M = −0.35, SE = 0.04, *p* = 0.002, *r* = 0.65) condition. Bias in the Imagery condition (M = −0.24, SE = 0.07, *p* = 0.11, *r* = 0.33) did not differ from the Baseline condition. An LMM (marginal *R*^*2*^ = 0.02; conditional *R*^*2*^ = 0.32) revealed that bias did not differ between the foveal item categories (χ^2^(2) = 0.07, *p* = 0.96), but it differed between the imagery conditions (χ^2^(1) = 4.59, *p* = 0.03): it was larger in the Passive Viewing than in the Imagery condition (MD = −0.11 ± 0.05, *t*(125) = −2.10, *p* = 0.04, *d* = 0.36). The interaction between category and condition was not significant (χ^2^(2) = 0.11, *p* = 0.95). The observed letter bias cannot be attributed to stimulus statistics, as letters and digits were fully counterbalanced across conditions, appearing with equal frequency as targets, flankers, and foveal items. Instead, the letter bias may be related to familiarity: Given that letters are encountered more frequently in reading and everyday visual tasks, participants may hold a stronger prior for letter stimuli. Under conditions of uncertainty, this prior could systematically bias responses toward letters, consistent with typical familiarity effects in perceptual decision making.^45^

### Experiment 2: Discrimination is enhanced by imagery of identical shape, irrespective of meaning

In Experiment 1, we found that imagining an item with the same shape and name as a crowded peripheral target increased target discriminability compared to an item with a different shape and name. This facilitation could be attributed to the shape of the foveal item. However, it remained possible that simply imagining the same letter name regardless of case provided a semantic cue. Thus, to disentangle shape and semantic content, we varied the letter case (shape identity) and the letter name (semantic identity) of the foveal item and the target in Experiment 2, using Lucida Handwriting font. We created four foveal item categories: “same-case same-name” (AA; for example, A and A), “same-case different-name” (AB; e.g., A and B), “different-case same-name” (Aa; e.g., A and a), and different-case different-name (Ab; e.g., A and b) (Figure 1B). If semantic cues drove the effect, then imagining an uppercase letter “A” should similarly help a lowercase “a” target (different-case same-name) as much as an uppercase “A” target. In contrast, if shape identity was critical, then only the same-case same-name (e.g., “A” and “A”) should improve performance. To anticipate our results, we found that only the “same-case same-name” (AA) condition gave a benefit, whereas “different-case same-name” (Aa) did not.

**Figure 3:**
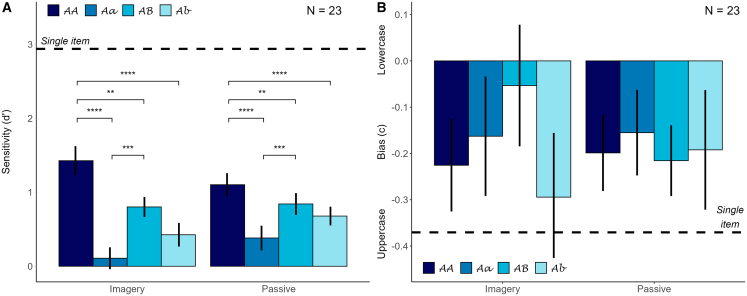
Results of Experiment 2 Sensitivity (A) and bias (B) results are shown across Imagery and Passive Viewing conditions for each foveal item category in Experiment 2. AA, Aa, AB, and Ab denote “same-case same-name,” “different-case same-name,” “same-case different-name,” and “different-case different-name” foveal item categories, respectively. Error bars represent standard errors of the mean. The dashed horizontal line indicates the average d′ and c for the single-item (i.e., Baseline) condition. Asterisks denote significant pairwise comparisons between conditions: ∗*p* < 0.05, ∗∗*p* < 0.01, ∗∗∗*p* < 0.001, and ∗∗∗∗*p* < 0.0001. Note that as the main effect of category was significant and no significant interaction was found, pairwise comparisons were conducted between categories, averaging over conditions.

The imagery conditions were the same as in Experiment 1: Imagery, Passive Viewing, and Baseline. Stimuli consisted exclusively of the letters (no digits as in Experiment 1) of the Lucida Handwriting font, approximately 1° in size and located at 8° eccentricity. The target and the flankers consisted of “A, B, E, H, N, R, T” uppercase and “a, b, e, h, n, r, t” lowercase letters. The foveal letter was either an uppercase or lowercase letter. Observers’ task was to indicate whether the target was an uppercase letter or a lowercase letter. Discriminability was calculated as in Experiment 1. Figure 3 displays the results of Experiment 2. As expected, observers’ discriminability was higher in the Baseline condition (M = 2.94, SE = 0.13) compared to the Imagery (M = 0.69, SE = 0.08, *p* < 0.0001, *r* = 0.88) and Passive Viewing (M = 0.75, SE = 0.09, *p* < 0.0001, *r* = 0.88), confirming the crowding effect.

An LMM with fixed effects of foveal item category (AA, AB, Aa, and Ab), imagery condition (Imagery and Passive Viewing), and their interaction, as well as a random intercept for participants (marginal *R*^2^ = 0.23; conditional *R*^2^ = 0.30) revealed a significant main effect of the foveal item category (χ^2^(3) = 53.43, *p* < 0.0001). The main effect of condition (χ^2^(1) = 0.35, *p* = 0.55) and the interaction between foveal item category and condition were not significant (χ^2^(3) = 5.51, *p* = 0.14).

Pairwise comparisons performed for the main effect of foveal item category showed that, irrespective of the imagery condition, discriminability was higher for AA foveal items compared to all other foveal item categories (AA and Aa: MD = −1.02 ± 0.15, *t*(168) = −6.88, *p* < 0.0001, *d* = 1.47; AA and AB: MD = −0.44 ± 0.17, *t*(168) = −2.99, *p* = 0.01, *d* = 0.64; AA and Ab: MD = −0.71 ± 0.20, *t*(168) = −4.81, *p* < 0.0001, *d* = 1.03). Additionally, discriminability was higher for AB foveal items compared to Aa foveal items (MD = −0.58 ± 0.14, *t*(168) = −3.89, *p* = 0.0006, *d* = 0.83). No significant differences were observed between the Aa and Ab categories, MD = 0.31 ± 0.16, *t*(168) = 2.07, *p* = 0.08, *d* = 0.44, or between the AB and Ab categories, MD = −0.27 ± 0.15, *t*(168) = −1.82, *p* = 0.08, *d* = 0.39.

We also analyzed the proportion of responses consistent with the foveal item’s category separately for foveal-target congruent and incongruent trials. Replicating the pattern observed in Experiment 1, foveal-consistent responses were high when the foveal item and peripheral target belonged to the same category (between 0.75 and 0.64 ± 0.15–0.11) and markedly reduced on incongruent trials (between 0.48 and 0.39 ± 0.14–0.10), indicating that response biases depended on foveal-target correspondence rather than a general tendency to report the foveal category.

Bias in all conditions was negative, showing a tendency to report “uppercase letter”. Wilcoxon tests showed that bias in the Baseline (M = −0.37, SE = 0.08) condition was larger than in the Passive Viewing (M = −0.19, SE = 0.07, *p* = 0.004, *r* = 0.61) and the Imagery (M = −0.18, SE = 0.11, *p* = 0.03, *r* = 0.46) condition. An LMM with an interaction effect (marginal *R*^*2*^ = 0.02; conditional *R*^*2*^ = 0.48) showed that category (χ^2^(3) = 2.32, *p* = 0.51), condition (χ^2^(1) = 0.01, *p* = 0.91), and their interaction (χ^2^(3) = 2.94, *p* = 0.40) were not significant. As in Experiment 1, the observed bias cannot be attributed to stimulus statistics since uppercase and lowercase letters were fully counterbalanced. To test whether scaling up a subset of lowercase letters (a, e, n, r) to match the height of the uppercase letters contributed to this bias, mean accuracies for each individual letter target were assessed. Proportion correct results showed that accuracy for lowercase letters—whether they were scaled up or not—was consistently lower than for uppercase letters.

Taken together, these results suggest that there is a benefit of identical “shape” (over identical “semantic”) imagery in reducing crowding: Imagery of an identical item (AA) improved discrimination compared to imagery of a semantically same item (Aa). The benefit of Passive Viewing of AA shows that performance can also be facilitated without imagery, which could be attributed to incidental cueing of the target 15 or the unfamiliarity and/or the complexity of the font used in Experiment 2 (see discussion). Interestingly, even AB proved more effective in reducing crowding than Aa, which could again be related to the processing of unfamiliar and complex letters used in this experiment (see discussion).

## Discussion

Our findings show how VMI can effectively improve basic spatial vision by reducing the impact of visual crowding. We demonstrated that imagining a central item identical to a peripheral target markedly enhanced target discrimination compared to imagining a different item or briefly viewing the central item without imagery (Experiment 1). The facilitation primarily depended on the shape of the imagined item rather than semantic cueing (Experiment 2). These findings suggest that visual imagery can produce perceptual signals strong enough to mimic those generated by actual stimuli, thereby enhancing visual object processing in cluttered environments.

Previous work has shown that crowding can be reduced by visual grouping of a peripheral target with a physically present foveal item.^15^^,^^46^ We hypothesized that imagining a central item could produce the same benefit without any physical stimulus. This idea is supported by findings showing that VMI can enhance target perception when the imagined and perceived stimuli match in content and location, likely because they rely on shared, analog visual representations.^2^^,^^3^^,^^8^^,^^47^ Consistent with this, imagining an identical foveal item (e.g., “A” with “A”) significantly improved peripheral discrimination compared to imagining a different item of the same category (e.g., “A” with “B”) or a different category (e.g., “A” with “7”). In Experiment 1, this facilitation was specific to imagery, as no benefit emerged during passive viewing. In Experiment 2, we found that only shape identity (same-case, same-name; AA) reduced crowding, whereas semantic identity (different-case, same-name; Aa) did not, indicating that precise visual matching, rather than semantic cueing, drove the effect. If semantic identity (letter name) were sufficient, we would expect AA and Aa trials to produce similar d′; instead, we observed a benefit only for AA, indicating a shape-specific mechanism. These results support the view that imagery engages early visual areas (V1–V3) in a retinotopic manner,^48^^,^^49^ recruiting the same neural circuits that process critical visual features –such as orientation, shape, spatial position to promote grouping across space. When an imagined foveal item matches a peripheral target, these shared resources facilitate perceptual grouping and enhance discrimination, even without concurrent visual input.

Importantly, although we emphasize the role of detailed visual representations in imagery, our findings do not imply that imagery operates exclusively at an early sensory level. Rather, we propose that imagery-generated representations interact with degraded peripheral input under crowding, enhancing the perception of the target when the sensory input is ambiguous. In this sense, imagery may engage early visual areas and strengthen the corresponding sensory representations.

A key question is whether visual imagery can enhance perception compared to typical conditions without imagery. In the present study, we addressed this question by comparing imagery conditions with passive viewing conditions, in which a central item was presented but not imagined, thereby approximating typical crowding paradigms in which no pre-cue is used. Thus, the Passive Viewing condition served as a baseline against which benefits due to imagery could be assessed. Results of Experiment 1 indicate that imagery improved performance: Sensitivity in the Imagery Identical condition (AA) was higher than in all three Passive Viewing conditions. Performance did not differ across the three Passive Viewing categories (Identical, Same Category, Different Category), showing that the foveal item (presented 4 s before the target) had no measurable effect on target discrimination. These results suggest that actively imagining the identical foveal item provided a distinct benefit compared to imagining no prior item, as in typical crowding experiments. Because the present design did not include a condition without any central item, we cannot determine whether imagery improves performance beyond standard uncued crowding. Similarly, the present design does not reveal whether imagining a target-incongruent item might impair performance. Future work, including such a baseline, will be needed to address these questions directly. Finally, in Experiment 2, variability across the Passive Viewing conditions suggests that even passively viewing a foveal item prior to the target stimulus influenced performance, potentially due to factors such as stimulus unfamiliarity and/or complexity, which are detailed later in discussion.

We found that passive viewing of the identical-shape item (AA) in Experiment 2 facilitated target discrimination compared to passive viewing of the identical semantic item (Aa). This result does not contradict Experiment 1; rather, it clarifies the scope and limits of improvement of visual discrimination by imagery: Imagery enhances perception when passive viewing does not already sufficiently pre-activate target-relevant representations. That passive viewing can itself engage relevant representations under certain conditions, such as complex or unfamiliar letters, is consistent with recent findings that peripheral sensitivity improves when identical stimuli are presented foveally.^15^

One possible explanation for the advantage of identical shapes in the Passive Viewing conditions is target cueing, where the foveal item initiates an attentional template to facilitate processing of matching targets. However, the long 4-s interstimulus interval (ISI) makes cueing an unlikely explanation: Classic studies typically report cueing benefits with much shorter ISIs of 150–300 ms.^50^^,^^51^^,^^52^ In a paradigm similar to ours, cueing benefits were only found at 200 ms, with only trends at 400–600 ms, indicating that target cueing alone cannot fully account for the facilitation in the Passive Viewing condition.^15^

A more plausible explanation is that the unfamiliar and visually complex font used in Experiment 2 required observers to attend to the distinctive visual features of each letter, including features distinguishing upper- and lower-case letters. This process may have involved the sustained activation of high-fidelity visual representations in short-term memory, akin to involuntary imagery, which could enhance peripheral discrimination when the foveal item matched the target in shape. Importantly, in this experiment, imagery did not provide measurable additional benefit beyond passive viewing, consistent with the idea that imagery is most effective when the relevant representation has not already been pre-activated. This account is consistent with findings using the same handwriting font, which showed that facilitation depended on a physical match between foveal and peripheral items (same name, same case), rather than semantic identity,^15^ demonstrating that facilitation arises from visual shape, not semantic identity. These results are consistent with recent models of foveal feedback, where feedback is proposed to preferentially strengthen foveal representations based on initially ambiguous feedforward inputs, while allowing peripheral-to-foveal interactions when delays or saccades occur.^43^^,^^44^ Imagining a central item in our study likely engages similar mechanisms, pre-activating foveal representations that help disambiguate peripheral targets under crowding, with the largest benefits occurring when passive viewing has not already engaged the relevant representations.

The unfamiliarity and complexity of the font used in Experiment 2 may also explain the facilitation observed for imagery of same-case different-name items (AB) compared to the imagery of identical semantic items (Aa). Unfamiliar fonts likely have less established visual representations in the brain compared to familiar fonts.^53^^,^^54^ Hence, when imagining a letter in such a font, observers may have relied more on its salient visual features (e.g., stroke style, curvature) rather than on stored representations. This could have improved performance more in the AB condition than in the Aa condition if salient imagined features were shared more frequently between items in the AB compared to the Aa condition. Although lower- and upper-case letters of the Lucida Handwriting font are relatively similar, notable differences exist: many upper-case letters contain similar vertical (e.g., B, E, H, N, R, T) and horizontal (e.g., A, E, H) strokes whereas lower-case letters often contain vertical strokes embedded in L-junctions with curved strokes (e.g., a, h, n, r) or parts of longer curved strokes (e.g., b, t), and clear horizontal strokes limited to the t (at a different location in the upper third). These structural differences may make upper-case features more salient and easier to rely on under uncertainty, also contributing to the observed bias toward upper-case responses. Future studies should directly test how familiarity and semantic identity interact during imagery, for example, by comparing familiar versus novel stimuli or by measuring which shape features observers rely on, to clarify the mechanisms underlying imagery-based facilitation in peripheral discrimination.

In summary, our study shows that VMI can reduce the impact of visual crowding, a phenomenon that impairs the ability to discern objects in cluttered environments. This suggests that under crowding, the target signal is not entirely lost but remains available—albeit degraded—for further processing. Crucially, we showed that mental imagery can enhance the degraded target signal through shape-based grouping (see also^15^), interacting with sensory input and modulating perception. By showing that imagining a central item identical to a peripheral target enhances target discrimination compared to imagining a different item or no imagery, we demonstrate that visual imagery can produce perceptual benefits akin to those provided by actual stimuli. This effect depends on the shape of the imagined item rather than its semantic content, suggesting that detailed, low-level visual processing is engaged during imagery. Together, these findings advance our understanding of how visual imagery interacts with perception, demonstrating that internally generated representations can improve spatial vision and influence visual experience.

### Limitations of the study

A limitation is that our study demonstrated these effects using letters and digits as stimuli, which provide a highly controlled but relatively constrained stimulus set. While this allowed precise manipulation of visual form and identity, future work should examine whether the same effects extend to more complex and naturalistic visual inputs, including complex stimulus classes and more ecologically valid viewing conditions. A further constraint is that the present study did not systematically manipulate the level of target-relevant activation provided by passive viewing, limiting the extent to which the influence of this factor on the magnitude of imagery benefits can be assessed. Future studies could directly test how varying levels of target-relevant activation during passive viewing modulate the magnitude of imagery benefits.

## Resource availability

### Lead contact

Further information and requests for resources should be directed to and will be fulfilled by the lead contact, Fazilet Zeynep Yildirim-Keles (fazilet.keles@bogazici.edu.tr).

### Materials availability

This study did not generate new unique reagents.

### Data and code availability

• The behavioral data generated during this study are deposited at OSF Data: https://osf.io/xafhs/ and are publicly available as of the date of publication.
• This paper does not report original code.
• Any additional information required to reanalyze the data reported in this paper is available from the lead contact upon request.

## Acknowledgments

This study was supported by funding from the 10.13039/501100001711Swiss National Science Foundation (grant no. PP00P1_163723) and the 10.13039/501100001665Agence Nationale de la Recherche (ANR-17-EURE-0017; ANR-10-IDEX-0001-02 PSL) to B.S.

## Author contributions

FZYK: Conceptualization, methodology, software, formal analysis, investigation, data curation, writing–original draft, writing – review and editing, and visualization. RA: methodology, formal analysis, review and editing, and visualization. BS: conceptualization, methodology, writing – review and editing, funding acquisition, resources, and supervision.

## Declaration of interests

The authors declare no competing interests.

## Declaration of generative AI and AI-assisted technologies in the writing process

During the preparation of this work, the author(s) used ChatGPT 4.o in order to improve language and readability. After using this tool/service, the author(s) reviewed and edited the content as needed and take(s) full responsibility for the content of the publication.

## STAR★Methods

### Key resources table

**Table:** 

REAGENT or RESOURCE	SOURCE	IDENTIFIER
**Deposited data**		

Experimental data (Experiment 1)	own	OSF Data: https://osf.io/xafhs/
Experimental data (Experiment 2)	own	OSF Data: https://osf.io/xafhs/

**Software and algorithms**		

R (v4.2.2)	R Core Team	https://www.r-project.org
RStudio (v2022.02.2)	RStudio Team	https://posit.co
G∗Power	Heinrich Heine University Düsseldorf	https://www.psychologie.hhu.de/arbeitsgruppen/allgemeine-psychologie-und-arbeitspsychologie/gpower
PsychoPy (v2.7.11)	Open Science Tools	https://psychopy.org/index.html

**Other**		

EyeLink 1000 eye tracker	SR Research	RRID:SCR_009602

### Experimental model and study participant details

We conducted a sensitivity power analysis^55^ using G∗Power 3.1^56^ for a 2 × 3 repeated-measures ANOVA with 24 participants. Assuming α = 0.05 and 95% power, the analysis showed that we were powered to detect a minimum effect of Cohen’s *f* = 0.27, equivalent to partial *η*^*2*^ ≈ 0.067. This corresponds to a medium effect, suggesting that the study is adequately powered to detect medium or larger effects.

A total of 24 healthy adult observers (15 females, 9 males; aged 20–32 years) participated in Experiment 1, including one of the authors (R.A.). In Experiment 2, 23 of the 24 observers (14 females, 9 males; aged 20–32 years) of Experiment 1 participated. All participants reported normal or corrected-to-normal vision and had no history of neuropsychological disorders, including dyslexia. All observers were native German speakers and participated either in exchange for course credit or voluntarily. Before the experiment, participants were informed about the experimental procedures and were made aware that they could withdraw from the study at any time without consequences. Except for the author, all participants were naive to the experimental hypotheses. Written informed consent was obtained from all observers prior to participation. The study adhered to the Declaration of Helsinki and was approved by the Ethics Committee of the University of Bern (2016-10-00008).

### Method details

#### Apparatus

Stimuli were generated using PsychoPy v2.7.11^57^ and displayed on a CRT monitor (1152 × 864 pixels) with a refresh rate of 110 Hz. Observers were seated 57 cm from the monitor while the gaze position of their dominant eye was tracked at a sampling rate of 1 kHz using an Eyelink 1000 (SR Research, Ottawa, Ontario, Canada). Viewing was binocular. Observers were supported by a head and chin rest to stabilize the head and minimize movements. Each block (baseline, imagery, and passive viewing) was preceded by a 9-point grid calibration followed by a 9-point grid validation. Trials with a gaze deviation ≥2° during target presentation were identified. Behavioral responses were collected using a standard computer keyboard. Experiments took place in a dimly lit room.

#### Stimuli

Either a single black disc (1 cd/m^2^, diameter = 0.2°) (in the Baseline condition) or two gray, vertically aligned discs (20 cd/m^2^, diameter = 0.2°, 1.5° spacing) (in the Imagery and Passive Viewing conditions) at the center of the screen served as fixation mark throughout the experiments. Two black, vertically aligned discs (1 cd/m^2^) were presented at either the left or right side of fixation to cue the target location.

In Experiment 1, the stimuli consisted of uppercase letters (A, B, F, G, K, P, R, Y) and digits (2, 3, 4, 5, 6, 7, 8, 9) presented in Arial font. All stimuli were black with a luminance of 1 cd/m^2^ against a gray background (42 cd/m^2^) and measured 1° in height, with widths varying slightly between 0.8 and 0.9° depending on the specific character. Stimuli were presented in two configurations: a single target (Baseline condition) or a target flanked by two items positioned to its left and right (Imagery and Passive Viewing conditions). The target was positioned on the horizontal meridian at 8° from fixation, either to the left or to the right. Flankers, when present, were positioned at a center-to-center spacing of 1.2° from the target. In each trial, the target was randomly selected from either the letter or digit set, with equal probability. The flankers always consisted of one letter and one digit, randomly positioned to the left or right of the target. The target and flankers never shared the same identity within a trial. In the Imagery and Passive Viewing conditions, an additional “foveal” item was displayed at the center of the screen. In the Baseline condition, no foveal item was presented. Foveal items fell into three categories: Identical, Same Category, and Different Category. In the Identical condition, the foveal item matched the target exactly (e.g., A and A). In the Same Category, the foveal item and target were different but from the same category (e.g., A and B, or 3 and 5). In the Different Category, the foveal item and target belonged to different categories (e.g., A and 3). The foveal item and flankers never shared the same identity within a trial. Foveal item categories were randomly distributed within a block, with 50% of the trials featuring items from the Different Category and the remaining 50% split evenly between the Identical and Same Category (25% each). This ensured that the foveal item was a letter in half the trials and a digit in the other half. Importantly, if observers simply relied on the foveal item to report the peripheral target’s category, Identical and Same Category items would yield equal performance benefits, as both would lead to the correct response; however, perceptually, identical cues can still facilitate target processing.^3^

In Experiment 2, uppercase (A, B, E, H, N, R, T) and lowercase (a, b, e, h, n, r, t) letters of Lucida Handwriting font served as stimuli. This font was chosen because of the relatively high similarity in curvature and complexity between uppercase and lowercase letters compared to other fonts, and as it has been successfully used in a previous study.^15^ However, some systematic differences between uppercase and lowercase remain with this font (see also discussion). All letters, including the small lowercase letters ‘a, e, n, r’, were set to 1° in height while the width slightly varied between 0.8 and 0.9° depending on the character. A larger font size was used for the letters ‘a, e, n, r’ to match their height to the other letters (resulting in slightly increased stroke width). The conditions (Baseline, Imagery, Passive Viewing) and target location (8°) were the same as in Experiment 1. When flankers were present, they were positioned at a center-to-center spacing of 1.38° from the target. In each trial, the target was randomly selected with equal probability from either the uppercase or lowercase letters set. The flankers consisted of one uppercase letter and one lowercase letter, randomly positioned to the left or right of the target, ensuring that the target and flankers never shared the same identity. Foveal items belonged to one of four categories: Same-Case Same-Name, where the foveal item matched the target in both case and name (e.g., a and a, or A and A), Same-Case Different-Name, where the foveal item matched the target in case but not name (e.g., a and b, or A and B), Different-Case Same-Name, where the foveal item matched the target in name but not case (e.g., a and A, or B and b), and Different-Case Different-Name, where the foveal item differed from the target in both case and name (e.g., a and B, or A and b). The foveal item and flankers never shared the same identity within a trial. The four foveal item categories were randomly presented within a block, with equal probability. All stimuli used in Experiment 2 are shown in the Figure S1.

#### Procedure

Baseline, Imagery, and Passive Viewing conditions were blocked. Observers were instructed to keep fixating at the center of the screen throughout all experiments while attending to the centrally and/or peripherally presented stimuli.

The Imagery condition started with a black fixation point presented for 1 s at the center of the screen. Next, the foveal item was presented for 1.5 s in between two central gray points, accompanied by two other black points in the periphery indicating the location of the upcoming target stimulus. The gray central points remained on the screen throughout the experiment while the peripheral cues stayed visible until the target disappeared. Observers were instructed to attend to the foveal item during its presentation. After the foveal item slowly faded out, observers were asked to imagine it for 4 s. The imagery duration was based on previous literature.^58^^,^^59^ After the imagery period, the target stimulus, along with two flankers, appeared for 0.15 s in between the two peripheral cues. Observers indicated whether the target was a letter or a digit in Experiment 1 and an uppercase or lowercase letter in Experiment 2 by pressing the corresponding key on the keyboard. In a second task, participants rated the similarity of the foveal imagined item and the target on a scale from 1 (not similar at all) to 3 (very similar). The next trial began 0.5 s after the response. The Passive Viewing condition was identical to the Imagery condition, except that observers were instructed to ignore the presented foveal item. The Baseline condition was similar to these conditions except that there was no foveal item, and no flankers presented. Experiment began with a black fixation point at the center presented for 1 s, followed by two peripheral cues presented for 0.5 s, then a target appeared in between the cues for 0.15 s. Observers indicated whether it was a letter or digit, and the next trial started 0.5 s after the response.

All observers participated in Experiment 1 first and then in Experiment 2. The block order (Baseline, Imagery, and Passive Viewing) was randomized for each observer. Before each block, observers completed practice trials until they felt comfortable with the instructions and task. In total observers completed 384 trials (2 experiments x 3 blocks × 64 trials). The trial combinations in each experiment and block were as follows. In Experiment 1, in the Imagery and Passive Viewing blocks, there were 32 trials with Different Category items, 16 trials with Identical items, and 16 trials with Same Category items while in the Baseline block there were 32 trials with letters and 32 with digits. In Experiment 2, in the Imagery and Passive Viewing blocks, there were 16 trials with each of the four foveal item categories (Same-Case Same-Name, Same-Case Different-Name, Different-Case Same-Name, and Different-Case Different-Name). In the Baseline block, there were 32 trials with uppercase letters and 32 with lowercase letters. In each block, a break was automatically given when half of the trials were completed. The whole experiment lasted approximately 2 h.

### Quantification and statistical analysis

All statistical analyses were conducted in R v4.2.2 (R Development Core Team, 2017, https://www.r-project.org/).

To evaluate the effect of experimental manipulations on performance, sensitivity (d') and bias (c) were calculated. For the calculation of d', signal trials corresponded to letters in Experiment 1 and uppercase letters in Experiment 2, and noise trials corresponded to digits in Experiment 1 and lowercase letters in Experiment 2. Correctly identifying a letter (uppercase letter) was classified as a hit, while failing to identify it was a miss. Similarly, correctly identifying a digit (lowercase letter) was a correct rejection, and failing to do so was a false alarm. The d' metric represents the sensitivity to discriminating letters (uppercase letters) from digits (lowercase letters), whereas the bias (c) reflects the response tendency: a negative bias indicates a predominance of letter (uppercase letter) responses, whereas a positive bias indicates a predominance of digit (lowercase letter) responses. To handle edge cases where hit rates or false alarm rates were 0 or 1, adjustments were made to avoid infinite z-scores. Specifically, a hit rate of 0 was replaced with 0.5/(*n*_signal trials_) and a hit rate of 1 was replaced with 1–0.5/(*n*_signal trials_). Similarly, a false alarm rate of 0 was replaced with 0.5/(*n*_noise trials_), and a false alarm rate of 1 was replaced with 1–0.5/(*n*_noise trials_).

Practice trials were not included to the analyses. Additionally, trials with gaze deviations ≥2° during target presentation were removed. On average, 62.80 ± 1.40 trials remained per participant and condition.

Prior to statistical testing, data were checked for assumptions of homoscedasticity, normality, and independence of residuals using diagnostic plots and statistical tests from performance package. As all assumptions were met, linear mixed models (LMMs) were used.

Sensitivity (d') and bias (c) values were analyzed using Wilcoxon signed-rank tests and Linear Mixed Models (LMMs). Wilcoxon tests were used to assess whether sensitivity was lower in crowded configurations (Imagery and Passive Viewing, averaged over all foveal item categories) compared to the single Baseline in order to determine if visual crowding occurred. LMMs were then used to evaluate whether imagery differentially affected performance in different foveal item categories. Since the Baseline condition did not include foveal-item categories, it was not included in the LMMs. A similar approach was used to analyze bias values: Wilcoxon tests examined whether bias differed between crowded and single conditions, while LMMs assessed the effect of imagery on bias within specific foveal item categories.

For LMMs, the data were modeled using the lmer function from the lme4 package. The models included condition and foveal item category as fixed effects, with observer as a random effect, allowing for random intercepts for each participant. The statistical models were defined as follows:

Model 1: d′ ∼ condition x foveal item category + (1|participant).

Model 2: c ∼ condition x foveal item category + (1|participant).

The statistical significance of fixed effects and their interactions in the LMMs was assessed using Type III Wald chi-square tests with an analysis of deviance table. For significant results of LMMs, post hoc comparisons were conducted using Holm corrections implemented in the emmeans package. Holm correction was also applied to paired Wilcoxon signed-rank tests.

Marginal and conditional *R*^*2*^ values were reported to assess the explanatory power of the LMMs. Effect size (*r*) for Wilcoxon test was calculated using the wilcox_effsize() function from the rstatix package. For linear mixed-effects analyses, effect sizes were calculated as Cohen’s *d* using model-based contrasts obtained from the linear mixed-effects model, standardized by the residual standard deviation of the fitted model.
