# Supplementary material for: improvement of peripheral visual discrimination through mental imagery
Source: iScience. 2026 Jun 17;29(7):116437. doi: 10.1016/j.isci.2026.116437 (PMC13310933; doi:10.1016/j.isci.2026.116437)
Supplement: Document S1. Figures S1 [file mmc1.pdf]

iScience, Volume *29*

Supplemental information

improvement of peripheral visual discrimination through mental imagery

Fazilet Zeynep Yildirim-Keles, Rahel Aschwanden, and Bilge Sayim

Supplemental information

Document S1. Figure S1

Same-Case Same-Name:

aa, bb, ee, hh, nn, rr, tt

AA, BB, EE, HH, NN, RR, TT

Same-Case Different-Name:

AB, AE, AH, AN, AR, AT

BA, BE, BH, BN, BR, BT

EA, EB, EH, EN, ER, ET

HA, HB, HE, HN, HR, HT

NA, NB, NE, NH, NR, NT

RA, RB, RE, RH, RN, RT

TA, TB, TE, TH, TN, TR

ae, ah, an, ar, at

ba, be, bh, bn, br, bt

ea, eb, eh, en, er, et

ha, hb, he, hn, hr, ht

na, nb, ne, nh, nr, nt

ra, rb, re, rh, rn, rt

ta, tb, te, th, tn, tr

Different-Case Same-Name:

Aa, Bb, Ee, Hh, Nn, Rr, Tt

aA, bB, eE, hH, nN, rR, tT

Different-Case Different-Name:

Ab, Ae, Ah, An, Ar, At

Ba, Be, Bh, Bn, Br, Bt

Ea, Eb, Eh, En, Er, Et

Ha, Hb, He, Hn, Hr, Ht

Na, Nb, Ne, Nh, Nr, Nt

Ra, Rb, Re, Rh, Rn, Rt

Ta, Tb, Te, Th, Tn, Tr

bA, eA, hA, nA, rA, tA

aB, eB, hB, nB, rB, tB

aE, bE, hE, nE, rE, tE

aH, bH, eH, nH, rH, tH

aN, bN, eN, hN, rN, tN

aR, bR, eR, hR, nR, tR

aT, bT, eT, hT, nT, rT

Figure S1. All stimuli used in Experiment 2.
